# Supplementary material for: Dimensionality reduction of diffusion MRI measures for improved tractometry of the human brain
Source: Neuroimage. 2019 Oct 15;200:89–100. doi: 10.1016/j.neuroimage.2019.06.020 (PMC6711466; doi:10.1016/j.neuroimage.2019.06.020)
Supplement: Multimedia component 1 [file mmc1.pdf]

Supplementary Data

Supplementary Table 1

Bundle	FA	AD	RD	MD	GA	Mode	AFD	AFD_{tot}	NuFO	FR
AC	0.45±0.14	8.1±1.2	5.9±1.3	8.0±0.9	0.82±0.28	0.68±0.43	0.44±0.18	0.21±0.05	1.4±0.5	0.21±0.08
AF (l)	0.47±0.13	7.5±1.4	5.3±0.9	7.3±0.4	0.90±0.28	0.51±0.50	0.46±0.20	0.25±0.05	1.7±0.7	0.29±0.09
AF (r)	0.46±0.14	7.3±1.5	5.2±0.9	7.1±0.4	0.86±0.29	0.49±0.52	0.44±0.20	0.25±0.05	1.8±0.7	0.28±0.09
CC	0.53±0.19	8.7±2.0	5.0±1.6	7.7±1.2	1.06±0.44	0.68±0.44	0.55±0.21	0.25±0.06	1.4±0.6	0.29±0.12
CST (l)	0.54±0.17	7.7±1.5	4.8±1.5	7.4±1.1	1.02±0.37	0.70±0.41	0.59±0.21	0.27±0.05	1.5±0.7	0.30±0.10
CST (r)	0.53±0.17	7.6±1.6	4.8±1.4	7.3±1.0	1.01±0.37	0.69±0.42	0.58±0.20	0.26±0.05	1.6±0.7	0.30±0.10
ILF (l)	0.47±0.13	8.1±1.4	5.6±0.9	7.8±0.6	0.89±0.28	0.57±0.47	0.42±0.18	0.23±0.05	1.5±0.6	0.24±0.09
ILF (r)	0.47±0.13	7.9±1.3	5.5±0.9	7.6±0.6	0.88±0.28	0.54±0.49	0.41±0.18	0.23±0.06	1.5±0.6	0.25±0.09
OR (l)	0.49±0.15	8.4±1.9	5.3±1.1	7.7±0.8	0.92±0.33	0.65±0.47	0.46±0.21	0.23±0.06	1.4±0.6	0.25±0.10
OR (r)	0.49±0.15	8.1±1.7	5.3±1.1	7.6±0.8	0.92±0.33	0.63±0.46	0.45±0.21	0.24±0.06	1.5±0.6	0.26±0.10
SLF (l)	0.42±0.14	7.2±1.3	5.5±0.9	7.2±0.5	0.81±0.28	0.43±0.52	0.38±0.18	0.24±0.07	1.8±0.7	0.26±0.09
SLF (r)	0.44±0.14	7.3±1.4	5.3±0.9	7.2±0.5	0.85±0.29	0.46±0.51	0.40±0.19	0.24±0.06	1.7±0.7	0.27±0.09
UF (l)	0.44±0.12	8.3±1.2	5.9±0.9	8.0±0.5	0.77±0.24	0.67±0.40	0.40±0.18	0.21±0.06	1.3±0.5	0.19±0.07
UF (r)	0.46±0.12	8.2±1.3	5.6±0.9	7.7±0.5	0.79±0.24	0.72±0.37	0.44±0.18	0.22±0.05	1.3±0.6	0.19±0.06
FAT (l)	0.44±0.15	7.6±1.4	5.5±1.0	7.4±0.5	0.82±0.31	0.52±0.48	0.41±0.20	0.23±0.07	1.6±0.7	0.25±0.10
FAT (r)	0.43±0.14	7.5±1.4	5.5±1.0	7.3±0.5	0.81±0.30	0.51±0.49	0.42±0.20	0.24±0.07	1.7±0.7	0.25±0.09
Cg (l)	0.48±0.15	8.3±1.5	5.4±1.0	7.7±0.5	0.91±0.32	0.67±0.44	0.47±0.20	0.22±0.05	1.5±0.6	0.26±0.09
Cg (r)	0.46±0.13	8.1±1.3	5.5±0.9	7.7±0.5	0.85±0.28	0.63±0.46	0.44±0.18	0.21±0.05	1.5±0.6	0.24±0.08
Genu	0.55±0.19	8.5±1.7	4.9±1.6	7.6±0.8	1.03±0.44	0.68±0.46	0.48±0.20	0.23±0.05	1.4±0.6	0.27±0.11
iFOF (l)	0.49±0.14	8.5±1.6	5.4±1.0	7.7±0.6	0.93±0.30	0.67±0.42	0.48±0.19	0.24±0.05	1.5±0.6	0.26±0.09
iFOF (r)	0.50±0.14	8.3±1.5	5.2±1.0	7.6±0.6	0.93±0.31	0.65±0.44	0.48±0.20	0.24±0.05	1.5±0.6	0.26±0.09
Splenium	0.61±0.21	9.6±2.1	4.5±2.0	7.8±1.4	1.25±0.52	0.78±0.39	0.56±0.22	0.25±0.06	1.2±0.5	0.32±0.13

Table 1: Quantitative overview of the group-averaged dMRI measures across all bundles (mean±std). AD, RD and MD are expressed in $mm^2/s \times 10^{-4}$.

Supplementary Figure 1

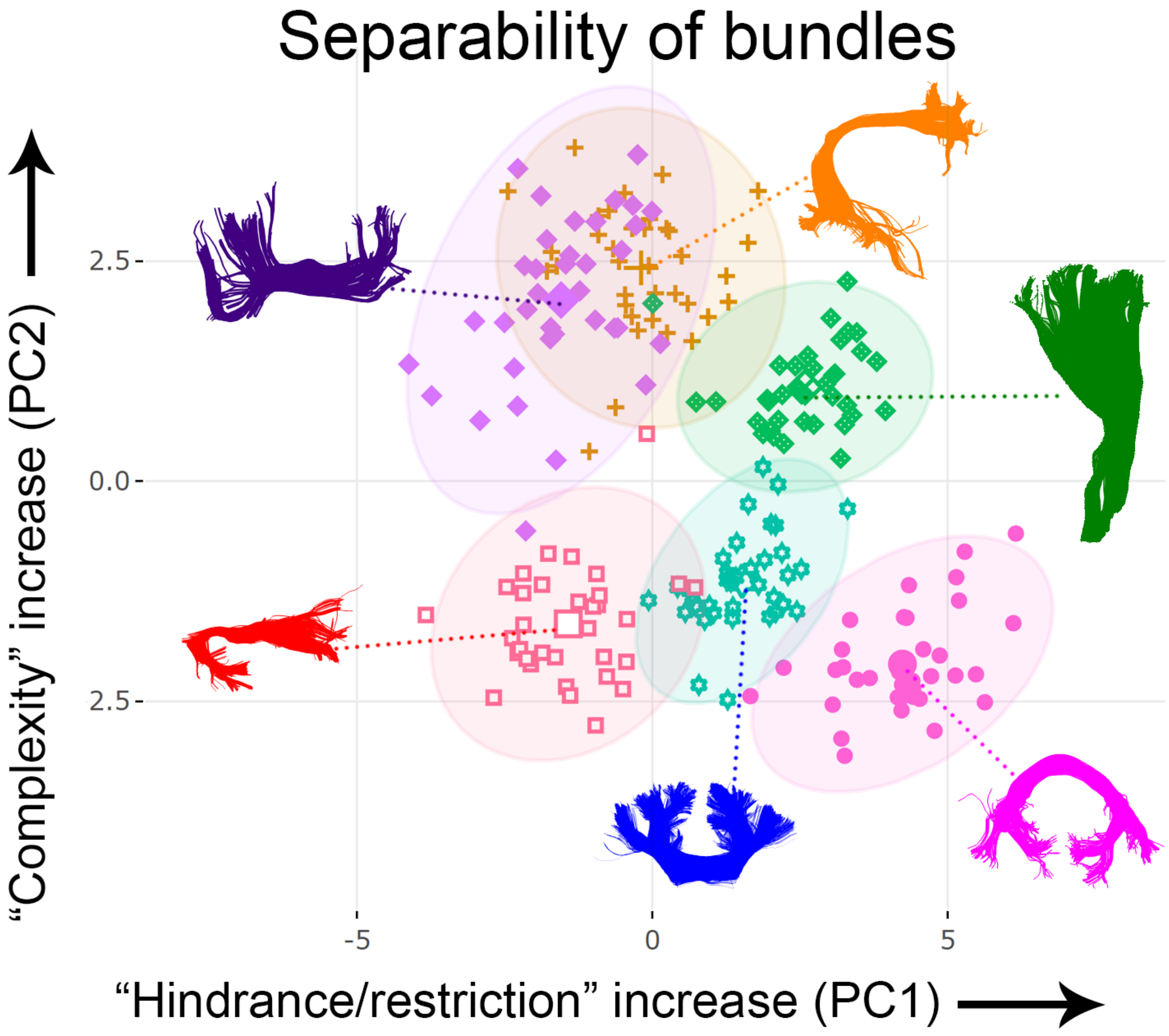

Figure 1: Supplementary Figure 1: Bundle clustering based on PC1 and PC2. The horizontal axis shows increasing restriction or hindrance perpendicular to the main axis of the bundles. On the right-most part of this axis are located densely-packed bundles such as the CST (green), genu (blue) and splenium (pink). The vertical axis represents the complexity degree of bundles (based on NuFO). On top of this axis are the arcuate fasciculus (orange) and superior longitudinal fasciculus (purple), two pathways which are known to have many crossing regions. Each point represents one subject. Concentration ellipsoids cover 95% confidence around the mean.

Supplementary Figure 2

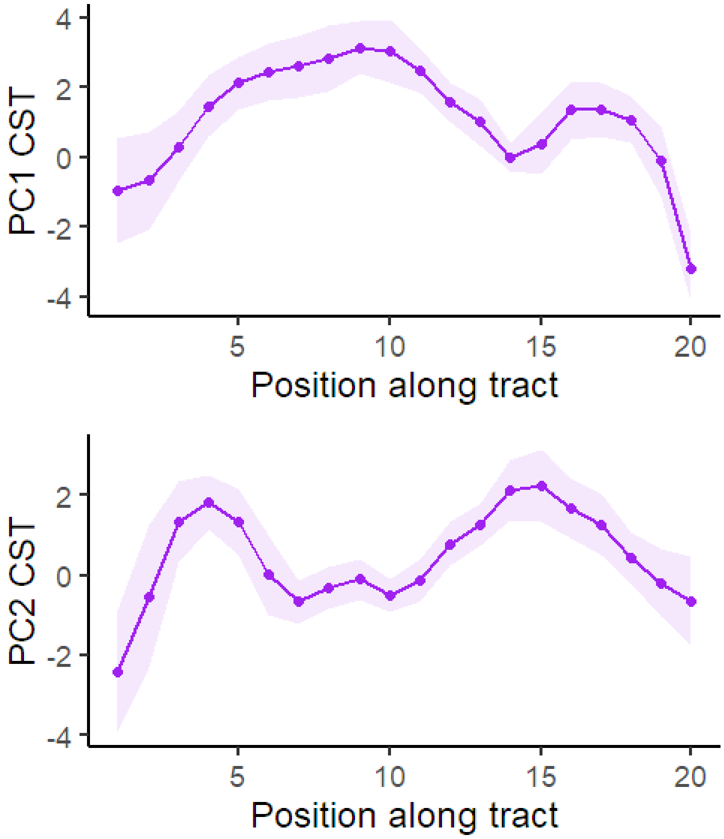

Figure 2: Supplementary Figure 2: Principal components profiled along the left CST. The profiles derived from PCA preserve the spatial heterogeneity of the input measures at different locations along the bundle. Shaded tract-profile area: ±1 standard deviation.

Virtual dissection scheme

The following details a brief description of the virtual dissection plan of each white matter bundle reported in the present study. All bundles were interactively extracted separately, in native space, repeating the method for each subject and hemisphere. Dissection were performed using FiberNavigator (Chamberland et al., 2014) by co-author KD, under the supervision of tractography specialists MC and DKJ.

Anterior commissure (AC) The AC is a thin white matter bundle which forms a direct connection between the temporal lobes (Wakana et al., 2007). It is located inferior to the most anterior part of the fornix and was captured by placing ROIs on the lateral branches in the ventral temporal lobe (Catani and De Schotten, 2008).

Arcuate Fasciculus (AF) To extract the AF, the first ROI was placed laterally to the corona radiata to capture the frontal-partial fibres. Then, a second ROI was placed posterolateral to the sylvian fissure to capture streamlines extending into the temporal lobe (Catani and De Schotten, 2008). An exclusion ROI was positioned below the curved portion of the bundle the remove spurious connections.

Mid-body of the Corpus Callosum (CC) Two ROIs were placed ventral to the location of the cingulum and medial to the lateral ventricles (one in each hemisphere) (Catani and De Schotten, 2008). Exclusion ROIs were used to exclude the Genu and Splenium (i.e., the anterior and posterior sections of the corpus callosum, respectively).

Corticospinal Tract (CST) A first ROI was placed at the level of the cerebral peduncle in the axial plane. A second ROI was placed over the central sulcus and capture fibres extending to the primary motor cortex (Chenot et al., 2018).

Inferior Longitudinal Fasciculus (ILF) The ILF is an association pathway forming a direct connection between the occipital and temporal lobe (Wakana et al., 2007). A first ROI was placed laterally, in the anterior temporal pole and a second ROI was placed in the posterior temporal lobe (Catani and De Schotten, 2008).

Optic Radiation (OR) To capture this bundle, a ROI was placed in the occipital lobe to encompass V1 and V2. A second ROI was planced anterolaterally to the geniculate body (Yamamoto et al., 2005; Chamberland et al., 2017).

Superior Longitudinal Fasciculus (SLF) For the SLF, a first ROI was placed on the superolateral aspect of the cingulum. Then a second ROI was placed over the supramarginal gyrus (Kamali et al., 2014).

Uncinate Fasciculus (UF) The UF connects the orbito-frontal cortex to the anterior temporal lobe. A first ROI was placed in the anterior part of the temporal lobe. A second ROI was positioned in the inferior medial frontal cortex

(Catani and De Schotten, 2008).

Frontal Aslant Tract (FAT) To extract this bundle, a selection ROI was placed over the superior frontal gyrus and a second one was placed middle of the inferior frontal gyrus (Catani et al., 2013).

Cingulum (Cg) The cingulum is a medial association fibre that is located over the corpus callosum. To extract this bundle, a first ROI was placed immediately postero-superior to the Genu. A second ROI was positioned in the posterior cingulate cortex (Catani and De Schotten, 2008).

Genu The genu is a commissural fibre that is the most anterior section of the corpus callosum. To extract this bundle, two ROIs were placed anterolateral to the most rostral portion of the corpus callosum in each hemisphere. This approach was used to capture the anteriorly arching fibres of the genu (Catani and De Schotten, 2008). An exclusion ROI was used to exclude streamlines extending posteriorly to the Genu, which make up the body of the corpus callosum.

Inferior Fronto-Occipital Fasciculus (iFOF) The iFOF connects the orbito-frontal cortex and the ventral occipital lobe. A first ROI was placed covering the anterior floor of the external capsule, and a second ROI was placed in the inferior part of the occipital lobe (Catani and De Schotten, 2008).

Splenium The splenium is the most posterior section of the corpus callosum, which joins the temporal and occipital lobes of the two hemispheres. Two ROIs were placed posterolateral to the most caudal section on the corpus callosum in the left and right hemisphere(Catani and De Schotten, 2008). An exclusion ROI was used to remove streamlines extending anteriorly to the splenium.

Catani, M., De Schotten, M.T., 2008. A diffusion tensor imaging tractography atlas for virtual in vivo dissections. cortex 44, 1105–1132.

Catani, M., Mesulam, M.M., Jakobsen, E., Malik, F., Martersteck, A., Wieneke, C., Thompson, C.K., Thiebaut de Schotten, M., DellâĂŹAcqua, F., Weintraub, S., et al., 2013. A novel frontal pathway underlies verbal fluency in primary progressive aphasia. Brain 136, 2619–2628.

Chamberland, M., Scherrer, B., Prabhu, S.P., Madsen, J., Fortin, D., Whittingstall, K., Descoteaux, M., Warfield, S.K., 2017. Active delineation of meyer's loop using oriented priors through magnetic tractography (magnet). Human brain mapping 38, 509–527.

Chamberland, M., Whittingstall, K., Fortin, D., Mathieu, D., Descoteaux, M., 2014. Real-time multi-peak tractography for instantaneous connectivity display. Frontiers in neuroinformatics 8, 59. doi:10.3389/fninf.2014.00059.

Chenot, Q., Tzourio-Mazoyer, N., Rheault, F., Descoteaux, M., Crivello, F., Zago, L., Mellet, E., Jobard, G., Joliot, M., Mazoyer, B., et al., 2018. A probabilistic atlas of the human pyramidal tract in 410 healthy participants. bioRxiv , 251108.

Kamali, A., Flanders, A.E., Brody, J., Hunter, J.V., Hasan, K.M., 2014. Tracing superior longitudinal fasciculus connectivity in the human brain using high resolution diffusion tensor tractography. Brain Structure and Function 219, 269–281.

Wakana, S., Caprihan, A., Panzenboeck, M.M., Fallon, J.H., Perry, M., Gollub, R.L., Hua, K., Zhang, J., Jiang, H., Dubey, P., et al., 2007. Reproducibility of quantitative tractography methods applied to cerebral white matter. Neuroimage 36, 630–644.

Yamamoto, T., Yamada, K., Nishimura, T., Kinoshita, S., 2005. Tractography to depict three layers of visual field trajectories to the calcarine gyri. Am. J. Ophthalmol. 140, 781–785.
